# Treatment of Diffuse Alveolar Hemorrhage: Controlling Inflammation and Obtaining Rapid and Effective Hemostasis

**DOI:** 10.3390/ijms22020793

**Published:** 2021-01-14

**Authors:** Jeong A. Park

**Affiliations:** Department of Pediatrics, Memorial Sloan Kettering Cancer Center, New York, NY 10065, USA; parkj2@mskcc.org or jeonga95@gmail.com; Tel.: +1-646-888-2320

**Keywords:** coagulopathy, corticosteroids, diffuse alveolar hemorrhage, hematopoietic stem cell transplantation, hemostasis, lung injury, pediatric, recombinant factor VIIa, rituximab, vasculitis

## Abstract

Diffuse alveolar hemorrhage (DAH) is a life-threatening pulmonary complication in patients with hematologic malignancies or systemic autoimmune disorders. Pathologic findings show pulmonary capillaritis, bland hemorrhage, diffuse alveolar damage, and hemosiderin-laden macrophages, but in the majority of cases, pathogenesis remains unclear. Despite the severity and high mortality, the current treatment options for DAH remain empirical. Systemic treatment to control inflammatory activity including high-dose corticosteroids, cyclophosphamide, and rituximab and supportive care have been applied, but largely unsuccessful in critical cases. Activated recombinant factor VII (FVIIa) can achieve rapid local hemostasis and has been administered either systemically or intrapulmonary for the treatment of DAH. However, there is no randomized controlled study to evaluate the efficacy and safety, and the use of FVIIa for DAH remains open to debate. This review discusses the pathogenesis, diverse etiologies causing DAH, diagnosis, and treatments focusing on hemostasis using FVIIa. In addition, the risks and benefits of the off-label use of FVIIa in pediatric patients will be discussed in detail.

## 1. Introduction

Diffuse alveolar hemorrhage (DAH) is a clinical syndrome characterized by acute onset of alveolar infiltrates and hypoxemia which result in progressive diffuse alveolar bleeding, requiring immediate treatment [1]. Many systemic diseases can cause DAH, but the pathogenesis is not well understood in the majority of cases, and the standard treatment for DAH has not been established even for the systemic autoimmune disorders. The treatment includes supportive cares such as correction of hemodynamic instability or coagulopathy and ventilatory support, high-dose corticosteroids, immunosuppressants, and plasmapheresis. Though, the overall mortality still remains high. To control systemic inflammation, subsequent cytokine storm, and life-threatening bleeding, multidisciplinary treatment is required. Although the role of systemic immunosuppressive treatment has been emphasized, systemic immunosuppressive treatment alone is not enough to stop the life-threatening bleeding, and it takes times to work: these single-disciplinary approaches often lead to treatment failure. 

Recombinant factor VIIa (FVIIa) was developed for the management of bleeding in hemophilic patients with inhibitors or in FVII-deficient patients. Despite this narrow indication, the off-label use of FVIIa has been increasing in non-hemophiliac patients to prevent or to treat uncontrolled bleeding in adults, children, neonates, and even preterm neonates [2,3]. The FVIIa has been reported to have a significant hemostatic effect in DAH as well [4,5,6,7]. However, there is no randomized controlled study to evaluate the efficacy in various conditions but also no consensus on the formulations, routes of administration, doses, and dosing regimens of FVIIa for the treatment of DAH. In addition, few studies have been reported in children. This review discusses the pathogenesis, pathologic findings, diagnosis and underlying etiologies of DAH, treatment strategies, hemostatic treatments including FVIIa, and the off-label use of FVIIa for DAH in adult and pediatric patients.

## 2. Pathogenesis of DAH

The DAH syndrome is attributed to the injury to the lung microvasculature including the capillaries, arterioles, and venules lining the alveoli. DAH can be clinically categorized into 4 major groups: immune associated [ANCA-associated vasculitis (AAV) and connective tissue disease], congestive heart failure associated, miscellaneous [infection, trauma, clotting disorder, drugs, malignancy, and hematopoietic stem cell transplantation (HCT)], and idiopathic. The major pathologic findings of DAH include antibody-mediated pulmonary capillaritis, bland alveolar hemorrhage, and diffuse alveolar damage [8,9,10]. Pulmonary capillaritis is the most common pattern and is defined by neutrophilic infiltration of the perivascular interstitium (alveolar septae) of the capillaries, endothelial edema, injury, and fibrinoid necrosis [9,11,12]. It is associated with systemic vasculitides, connective tissue disorders, immune complex mediated disorders, and post-transplant hemorrhages, and systemic immunosuppressive treatment is effective to control DAH [12]. Hemosiderin-laden macrophage accumulation is characteristic in 24–48 hours after the initial vessel injury [8,13]. Bland alveolar hemorrhage is not directly linked with inflammation or destruction of the alveolar capillaries, venules, and arterioles but with widespread leaking of red blood cells (RBCs) into the alveoli [8,10]. It is observed in drug-induced DAH, systemic lupus erythematosus (SLE), disseminated intravascular coagulation (DIC), cardiac originated DAH (mitral stenosis or mitral regurgitation), and infections such as HIV or infective endocarditis [8,9]. Generally, systemic immunosuppressants are not indicated except in cases of systemic autoimmune disease. Diffuse alveolar damage, the primary lesion in acute respiratory distress syndrome (ARDS), is characterized by interstitial and intra-alveolar edema, capillary congestion, microthrombi, epithelial necrosis and sloughing, the presence of fibrinous exudates in alveolar air spaces, and hyaline membrane formation [14]. It is associated with ARDS, cytotoxic drugs, radiation treatment, SLE, and cocaine inhalation [15,16].

Systemic inflammation involving pulmonary vasculature triggered by overactive autoimmune response and cytokine storm deemed to be the primary cause of DAH. Macrophage activation and high ferritin and IL-6 levels have been implicated in the pathogenesis of acute lung injury, inflammation, and pulmonary hemorrhage [17]. Pulmonary capillaritis-associated DAH bears on fibrin thrombi occluding the intra-alveolar capillaries and fibrinoid necrosis of the small blood vessels. It is often accompanied by inflammation of large blood vessels, IgG/C3 deposition in the alveolar walls, and erythrocytes extravasation into the alveolar spaces [18]. In animal models, DAH is mediated by opsonin-dependent uptake of dead cells by natural IgM and subsequent activation of the early classical complement pathway [19]. During inflammation, bone marrow-derived macrophages are recruited to the alveoli and differentiate into proinflammatory (M1-like) macrophages [20], and the IgM/C3-opsonized dead cells engage CR3/CR4 (a component of C3b receptor 3 and 4) on macrophages, expediting the development of DAH [19]. Neutrophil extracellular traps (NETs), fibrous networks which protrude from the membranes of activated neutrophils, also have a detrimental role in both autoimmune disorders and acute lung injury [21]. NETs accelerate the inflammatory processes by releasing a wide range of active molecules like danger-associated molecular patterns (DAMPs), histones, active lytic-enzymes, and multiple cytokines in the extracellular space [22]. Imbalance between NETs formation and degradation exacerbates immune responses and tissue injury [23], and prolonged exposure to NETs-related cascades increases systemic organ damage [24]. NETs are closely associated with development of DAH in murine autoimmune disease models, and targeting NETs with DNase-I reduced severity of DAH lesions and improved survival [25,26].

DAH after HCT is also closely associated with lung injury and subsequent vasculopathy. High-dose chemotherapy, thoracic or total body irradiation, and undocumented infections have been implicated as the initial injury to the lung leading to post-transplant DAH [27]. Animal models suggest that alveolitis develops during the acute phase of graft-versus-host disease (GVHD), and it is characterized by alveolar hemorrhage, increase in the alveolar leukocytes, platelet microthrombi, damage of alveolar endothelial and epithelial cells, increased turnover rate of alveolar cells, and increased cell counts and protein concentration of the bronchoalveolar lavage (BAL) fluid [28,29]. Irrespective of post-transplant leukopenia, neutrophils and neutrophil products are detected in the lower respiratory tract of HCT recipient at the time of DAH [30]. Besides, vasculitis of small muscular arteries and thrombotic microangiopathy in the form of endothelial swelling and thrombi are frequently observed in acute hemorrhagic pulmonary edema after transplantation [9,31]. The vasculopathy manifests as concentric intimal or medial hyperplasia with luminal narrowing, prominent myxoid changes, extravasated RBCs, and the presence of foamy histiocytes apart from thrombotic microangiopathy [31,32,33]. Add on, hematopoietic growth factors (G-CSF) and cytokine storms make worse the alveolar damage and capillary leakage by increasing neutrophil infiltration into the lungs [30]. Dysregulated cytokine release including both TH1 (IL-2, IL-6, IFN-γ, and TNF-α) and TH2 (IL-9 and IL-15) cytokines further potentiates the inflammatory response [34]. 

## 3. Diagnosis of DAH

The differential diagnosis of DAH is broad, and the etiologies of DAH can be broadly divided into immune- and non-immune-mediated causes (Table 1) [35]. A careful history, physical examination, and laboratory tests can often help to establish the risk factors and most likely etiology of the alveolar hemorrhage. Bronchoscopy is the key investigation needed to diagnose DAH by lavage and to exclude other associated infections. Bronchoscopy has higher yield if performed within the first 48 h. Persistent or increasing blood on three sequential lavage aliquots from one affected area of lung supports the diagnosis of DAH [36]. The number of hemosiderin-laden macrophages should be counted in cases of subacute or recurrent DAH [8,16]. The finding of ≥20% hemosiderin-laden macrophages in BAL fluid is commonly regarded as diagnostic of DAH and closely associated with severity [36,37,38]. BAL specimens should be sent for routine bacterial, mycobacterial, fungal, and viral cultures, and Pneumocystis stains to exclude infections. The role of transbronchial lung biopsy is not established in the diagnosis of DAH as the area of involvement is often patchy [8], but in any patients presenting with DAH of unclear causes a lung biopsy is strongly recommended to determine the underlying etiologies [10,39,40]. Imaging studies including chest radiographies and high-resolution chest computed tomography (CT) scans provide additional information to support a diagnosis of DAH. Typical pattern of DAH includes focal or diffuse areas of ground glass opacities or consolidations as a consequence of alveolar filling. Laboratory tests include a complete blood count with differential; coagulation studies; serum BUN and creatinine analysis; ANCA testing (c-ANCA and p-ANCA); antigen-specific ELISA [proteinase 3(PR3) and myeloperoxidase (MPO)], analysis of anti-phospholipid antibodies (APL), lupus anticoagulant, anti-cardiolipin (CL) antibodies, anti-β-2 glycoprotein1 (β2GP) antibodies, anti-glomerular basement membrane (GBM) antibodies, anti-nuclear antibodies (ANA), and rheumatoid factor (RF); urinalysis with urine sediment assessment; and a urine drug screen [9,41]. 

## 4. Treatment of DAH

DAH, especially acute macroscopic hemorrhage, has a high mortality rate, requiring prompt and aggressive multidisciplinary management. Treatment for DAH involves three major disciplines: (1) supportive care including hemodynamic correction, transfusion, and ventilator support, ranging from oxygen supplementation to mechanical ventilation with high positive end-expiratory pressure (PEEP) producing a tamponade effect to limit capillary bleeding; (2) treatment of underlying disease including intensive immunosuppressive treatments to control disease activity, plasmapheresis to remove autoantibodies, and antivirals or antibiotics for infection-associated pulmonary hemorrhages; and (3) rapid and effective local hemostasis [7]. 

The most immediate life-threatening complication of DAH is acute hypoxemic respiratory failure. When severe DAH results in ARDS, high levels of FiO_2_ and PEEP are often needed to achieve acceptable oxygenation. Although there is no consensus on ideal PEEP levels for DAH and it should be adjusted by the severity of respiratory failure and lung recruitability, DAH has been managed with high PEEP and permissive hypercapnia to reduce the active bleeding and prevent lung collapse [42]. The PEEP used in severe ARDS lies around 8.5 cm H_2_O [43], but the PEEP level should be selected by carefully considering oxygenation advantage and the putative benefits on lung protection [44].

To control the inflammatory activity, high-dose corticosteroids are recommended to start promptly, along with treatment for underlying disease. Corticosteroids have been accepted as a mainstay of treatment aimed at reducing acute inflammatory responses such as lung alveolar epithelial swelling, thrombotic microangiopathy, and increased inflammatory cells and cytokines [10,40,45]. Based on anecdotal reports and retrospective studies, systemic high-dose corticosteroids (500 mg to 2 g/day or 30 mg/kg/day of intravenous (iv) methylprednisolone for 3–5 days followed by gradual tapering over 4 weeks) are recommended to treat DAH [4,40,46,47]. However, steroid treatment alone is not sufficient to stop acute macroscopic pulmonary bleeding and is often fatal if the etiology is infectious or if the patient is in an immunocompromised status; the benefit of high-dose corticosteroids in critically ill patients remains undefined. Despite the widespread use of high-dose corticosteroids for DAH, the mortality exceeds 50%, especially in patients requiring intensive care unit (ICU) admission or in patients received HCT [48]. For the treatment of AAV, reduced-dose glucocorticoids had comparable efficacy to standard-dose corticosteroids with respect to mortality or incidence of end-stage renal disease (ESRD) but also reduced the incidence of serious infections at 1 year [49]. A study investigating the dose effect of corticosteroids for DAH suggested that patients treated with low-dose methylprednisolone (<250 mg/day) had a significantly lower ICU mortality rate compared to the patients treated with medium-dose (250–1000 mg/day) or high-dose (>1000 mg/day) methylprednisolone, and overall mortality did not differ by the doses of corticosteroids, raising questions about the validity of high-dose corticosteroids for DAH [48].

To eliminate autoreactive antibodies or triggering factors rapidly, plasmapheresis has been used as an adjunctive therapy particularly in autoimmune connective disorders, such as anti-GBM disease, AAV or SLE-associated pulmonary capillaritis. Plasmapheresis is performed daily or on alternating days for 14 days. Each exchange involves 1–1.5 times the total plasma volume and is replaced with albumin or FFP. Although retrospective studies reported excellent patient outcome and safety [50], long-term follow-up data or matched analyses to compare the efficacy of plasmapheresis to methylprednisolone have failed to show significant benefit [51,52,53]. The evidence-based guidelines of the American Society for Apheresis recommend using plasmapheresis in AAV patients with DAH presenting with hypoxemic respiratory failure requiring either high-flow supplemental oxygen or mechanical ventilation [54]. Recently released data of randomized controlled trial of plasma exchange and glucocorticoids for treatment of AAV (NCT00987389) suggested that the use of plasmapheresis did not reduce the mortality or the incidence of ESRD [49].

Rituximab, a chimeric monoclonal antibody targeting CD20, has been used as another viable alternative. Antibody-mediated modification and/or depletion of ANCA producing CD20(+) plasma cells is a proposed mechanism for rituximab to decrease autoantibody production and to control disease activity [55,56]. Although most of the studies have been limited, they suggest that rituximab is an effective therapeutic option for DAH in connective tissue disease or autoimmune disorders. Rituximab on a compassionate use basis for patients with refractory AAV reported successful treatment outcomes [57,58,59,60]. These encouraging results led to randomized controlled trials evaluating the effect of rituximab as a remission induction therapy in patients with severe AAV (NCT00104299, NCT01731561). Comparison between rituximab and cyclophosphamide pulse therapy for AAV showed a similar efficacy [61]. A randomized trial comparing the effect of combination therapies of glucocorticoids plus rituximab (375 mg/m^2^ once weekly for 4 weeks) and glucocorticoids plus cyclophosphamide (2 mg/kg/day) also demonstrated comparable efficacy of rituximab for the remission induction in severe AAV, and rituximab showed a better effectiveness for recurrent disease, major renal disease, or alveolar hemorrhage [58]. For long-term remission, rituximab was compared to azathioprine maintenance (NCT00748644), and rituximab treatment showed significantly longer remission duration and improved overall survival [61]. 

**Figure 1:**
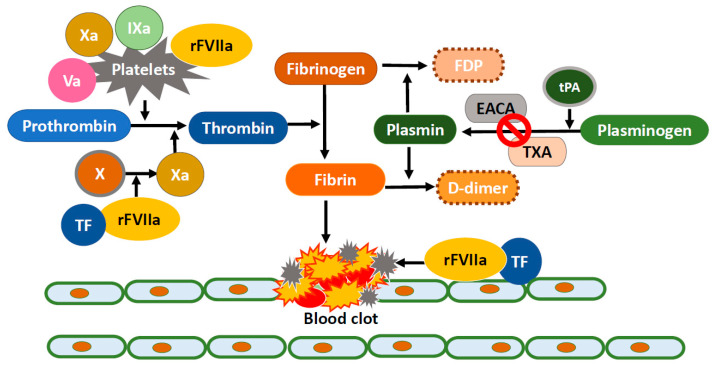
Coagulation and fibrinolytic cascades and the mechanisms of hemostatic agents.

For obtaining rapid hemostasis, coagulopathy should be closely monitored and quickly corrected. Commonly accepted targets are platelet counts more than 50,000/μL and a prothrombin time-international normalized ratio (PT-INR) less than 1.5. Depending on the causes, platelet transfusions, vitamin K supplementation, cryoprecipitates, and fresh frozen plasma should be supplemented. Besides, in order to stop the bleeding, various prothrombotic treatments including antifibrinolytics, particularly lysine analogues tranexamic acid (TXA) and epsilon aminocaproic acid (EACA), thrombin, and FVIIa have been used for DAH and reported with a variety of success rates. Mechanisms of the hemostatic agents are described in Figure 1.

TXA prohibits conversion of plasminogen into plasmin inhibiting fibrinolysis and stabilizes blood clots [62]. In addition to intravenous use, intrapulmonary or aerosolized TXA has been used for DAH [63,64,65]. Nebulized TXA led to complete or near cessation of bleeding in 10/18 pediatric patients with DAH [65], and recently, Neil et al. published a successful treatment outcome in 19 pediatric patients with DAH: 18 out of 19 patients had stopped bleeding after TXA inhalation [64]. However, TXA therapy failed to show significant effect on reducing bleeding-associated mortality in patients with hematologic malignancies [66] but also increased risk of post-operative seizures [67] and showed limited efficacy on profound and recurrent bleedings [65]. EACA, another option for hemostatic treatment, is a lysine analog that binds competitively to plasminogen, blocking plasminogen from binding to fibrin and the subsequent conversion to plasmin, resulting in the inhibition of fibrin degradation (fibrinolysis) [68]. EACA has been used in addition to corticosteroids in patients with post-transplant DAH and reported to reduce 100-day mortality rate from 83% to 44% in patients treated with corticosteroids [69]. Though, a recent follow-up study reported that adjuvant treatment with EACA did not yield a significant difference in the outcomes of patients with DAH after HCT. The overall mortality on day 100 was still high at 85% [48]. 

In addition, the effects of thrombin or fibrinogen-thrombin for treatment of severe hemoptysis have also been reported [70,71]. Recently, Lee et al. published the efficacy of intrapulmonary thrombin treatment for DAH in patients with hematologic malignancies. Intrapulmonary thrombin stopped bleedings rapidly in 13 of 15 patients’ refractory to corticosteroids with TXA treatment and significantly improved oxygenation status without thromboembolic complications [72]. Thrombin production is the final coagulation step required to convert fibrinogen to fibrin which produces a hemostatic lattice for platelet aggregation and thrombus formation at the site of injury [73]. Besides the coagulation cascade, thrombin causes vasoconstriction at the smooth muscle cell level and promotes platelet aggregation at the site of the thrombus [74].

## 5. Recombinant FVIIa Treatment for DAH

Recombinant FVIIa (FVIIa) is also used as an alternative ‘broad spectrum hemostatic agent’ to enhance hemostasis in patients with life-threatening intractable bleeding. Given that tissue factor (TF) pathway inhibitors (TFPI) increased in inflamed alveoli and these TFPIs prevent FVIIa-TF formation and FX activation, the inflamed lungs are more susceptible to bleeding [75]. FVIIa can overcome the effect of TFPI and restore thrombin generation. It promotes hemostasis via both a TF-dependent pathway at the sites of endothelial injury and a TF-independent pathway which directly activate factors IX and X on the surface of activated platelets in the absence of TF (Figure 1). Factor X converts prothrombin to thrombin, which in turn converts fibrinogen to fibrin. Alveolar TF remains high in inflammatory pulmonary conditions, including DAH, acute respiratory distress syndrome (ARDS), and pneumonia, as well as after lipopolysaccharide local challenge in the alveoli [5,76,77,78]. Alveolar TF-FVIIa complex activates coagulation factors IX and X, which in turn initiates a cascade of reactions, leading to thrombin burst and fibrin formation at the site of injury [5]. However, due to the separation between the alveolar and systemic compartments of the lung, FVIIa, similarly to most biologics, requires a high systemic concentration to affect specific receptors in the alveolar compartment. This requires higher and repeated doses of intravenous FVIIa, associated with a higher risk of thromboembolic complications [7,79,80]. On the other hand, intrapulmonary locally administered FVIIa has been reported to have successful outcomes with a relatively small dose (50 μg/kg) and less frequent administration [5,81,82]. Intrapulmonary (ip) administration can ensure that FVIIa reaches its alveolar receptor, TF, and it decreases systemic adverse effects because the alveolocapillary membrane does not allow the transmembranous passage of FVIIa. Pulmonary-administered FVIIa combines with TF and forms TF-FVIIa complex which activates coagulation factor IX and X, inducing balanced hemostasis. With relatively small doses, FVIIa results in both durable hemostasis and a significant improvement in oxygen transport capacity [7]. Moreover, TFPIs produced by alveolar macrophages are highly expressed in the airspace in inflammatory conditions secondary to acute lung injury and contribute to the balanced hemostasis without the theoretical risk of intra-alveolar thrombotic complications with intrapulmonary FVIIa [7,83,84]. Accordingly, treatment with FVIIa has a potentially high benefit-to-risk ratio in DAH when administered via the local intrapulmonary route. 

Case reports and clinical studies have reported the effectiveness of FVIIa in various conditions, including thrombocytopenia, functional platelet defects, hemorrhagic complications after HCT (e.g., DAH and hemorrhagic cystitis), gunshot wounds, and coagulopathy of liver failure [85,86,87,88,89,90,91,92]. However, the efficacy and side effects of FVIIa in non-hemophiliac settings are largely anecdotal, and many questions still remain regarding the appropriate indications and guidelines for use, risks of thrombotic complications, monitoring, dosing, and integration with transfusion therapy. A Cochrane review of the off-label use of FVIIa reported that FVIIa did not have a significant mortality benefit over placebo but showed a trend toward control of bleeding, a lower number of transfusions, and arterial thromboembolic events [93]. 

There is growing literature supporting the use of FVIIa to stop acute pulmonary hemorrhage following various diseases and injuries, including pneumonia, HCT, metastatic cancer, idiopathic pulmonary hemosiderosis (IPH), and immune-associated vasculitis. Due to lack of randomized controlled studies, FVIIa has been used as a final effort to stop the bleeding, and many studies have reported the successful use of FVIIa for life-threatening DAH [5,81,82,94,95]. Although the optimal dose and dosing intervals remain to be determined, systemic administration usually entails the intravenous administration of 35–200 μg/kg as either a single dose or repeated doses every 2–4 h [4,6,95,96], and intrapulmonary therapy typically involves bronchoscopy with a total dose of 50–90 μg/kg of FVIIa diluted in normal saline as either a single dose or, if bleeding continues, as repeated doses over 24 h [7,81,97,98]. The FVIIa treatment for pulmonary hemorrhage in adults is listed in Table 2. Twenty-nine in 111 cases were related to immune disorders, and the remaining 82 cases were non-immune-related DAH where hematopoietic stem cell transplant (HCT)-related DAH showed a majority (75%, 62 cases). While the mortality of immune-mediated DAH was 25% (7 in 28 patients), that of non-immune-mediated DAH showed a higher mortality rate, 47% (27 in 58 patients: the outcome for the other 24 patients was not specified). Although the mean dose of FVIIa for iv injection was 427 µg/kg/episode for immune-related DAH and 167 µg/kg/episode for non-immune-related DAH, most of the fatality cases were not directly related to DAH, and the complete response rate was similar in both groups; 19 in 21 patients and 23 in 23 patients. For iv use, approximately 250 µg/kg/episode of FVIIa was administered, while, 50µg/kg/episode of FVIIa was used for ip administration. The response rate did not differ by the administration route or administered dose: 42 in 44 patients with iv FVIIa and 19 in 21 patients with ip FVIIa showed complete response.

## 6. Recombinant FVIIa Treatment for DAH in Children 

The off-label use of FVIIa in pediatric patients has also been expanding despite the absence of adequate studies addressing safety and efficacy. According to a multi-center cohort study, 83% of FVIIa use was off-label, and the off-label use of FVIIa increased 10-fold between 2000 and 2007 [114]. The Haemostasis Registry reported the results of the off-label use of FVIIa in 388 children [115]. The median age was 12 months, and the medians of the first dose and total dose were 114 μg/kg and 1.2 mg, respectively. They observed a reduction in the number of transfusions after the use of FVIIa (*p* < 0.01) and a subjective response rate of 82%; thromboembolic complications were reported in 5.4% to 10.9% of the patients, which were similar to the published incidence in adults [79,116,117]. These results suggest that FVIIa is an effective hemostatic agent in severely ill pediatric patients experiencing life-threatening hemorrhage, and FVIIa can reduce blood product requirements [2,115,118]. However, its benefit for neonates and infants less than 1 year of age remains unclear. Young et al reported that neonates had a lower response rate of 47% and a higher risk of thrombosis (17.6%) than older aged groups (more than 1 month) [118]. The SeveNBleeP registry, a web-based registry of FVIIa use in non-hemophiliac pediatric patients including 42 infants less than 1 year reported that there was no significant reduction in requirements for blood products after FVIIa treatment and no significant increase in thromboembolic complication (2.4%) [2]. The FVIIa has a shorter half-life and more rapid clearance in children compared with adults; therefore, higher and more frequent doses of FVIIa are often required to control significant bleeding in children, especially in neonates and infants [2,115,118]. Considering the relatively poor results of FVIIa and the low levels of natural anticoagulants (proteins C and S and antithrombin-III), the risk of thromboembolic complication can increase in neonates and infants, and the risk-to-benefit ratio can be higher, requiring careful decision.

Many causes such as perinatal asphyxia, very low birth weight (VLBW), mechanical ventilation, respiratory distress syndrome (RDS), exogenous surfactant therapy, sepsis, hypothermia, patent ductus arteriosus, and coagulopathies can trigger pulmonary hemorrhage in pediatric patients including neonates [3,119]. The FVIIa treatment for pulmonary hemorrhage in pediatric patients is listed in Table 3. Data on 45 children are available, the majority were classified as non-immune related DAH, and only 3 cases were immune related DAH. Among 33 pediatric cases with past history information, 16 cases were associated with hematologic malignancies, 5 cases were associated with HCT, 4 cases were preterm, and 5 cases were related to cardiovascular disease. The overall mortality was 30% (10 of 33 cases with detailed information), and the pediatric patients with hematologic malignancies showed a high mortality (9 in 16 patients, 56%). Complete response after treatment of FVIIa was observed in 64.4%, and a partial response (bleeding improved but not ceased) was in 24.4%, with 11.1% having no response to treatment. Two thromboembolic complications were reported (4.4%) [118,120]. Among 45 cases, 29 patients received iv rFVIIa and 16 patients received ip rFVIIa treatment. The mean dose of rFVIIa was 400 µg/kg per episode (range, 60–3150 μg/kg) for iv treatment and 46 µg/kg per episode for ip treatment, respectively. However, the outcome for ip rFVIIa was not inferred to iv rFVIIa: among 29 patients who received iv rFVIIa, 15 achieved complete response, 9 showed partial response, and 5 had no response (82% of response rate); among patients who received ip rFVIIa, 14 had complete response and 2 had partial response (100% of response rate). The patients who received iv FVIIa required multiple doses to achieve satisfactory hemostasis. Although these data cannot represent the effect of FVIIa for DAH in pediatrics, iv FVIIa appears to be less effective for DAH than other life-threatening bleedings [95,118]. Due to pharmacokinetic characteristics of children, higher and multiple doses of FVIIa may be needed. On the other hand, the patients treated with ip FVIIa showed a significant improvement in their oxygenation capacities (PaO2/FiO2 ratio) on subsequent days after FVIIa treatment [89,99], and one child experienced thrombotic obstruction in his endotracheal tube (ETT); there was no evidence of intra-alveolar thrombotic deposition (hyaline membrane formation) [120]. We experienced successful immediate hemostasis after treatment of ip FVIIa in pediatric patients with refractory severe DAH which did not respond to other treatments including TXA, high-dose corticosteroids, rituximab, and multiple transfusions [89]. These data showed that pulmonary hemostasis can be induced more easily from the alveolar side than from the endothelial side with reduced systemic risk of complication, suggesting intrapulmonary administration of FVIIa as an effective strategy for DAH in children as well as in adults.

## 7. Conclusions

DAH should be suspected in any patient with alveolar infiltrates on chest radiographs, hypoxemia, anemia, and hemoptysis. DAH is a clinical syndrome that can be a manifestation of multiple different etiologies, and identifying the underlying etiology is important to determine treatment strategy. In life-threatening DAH, rapid and effective hemostasis along with the appropriate treatment for the underlying disease contributes to patient survival. Considering the substantial risk of thromboembolic complications with the use of a large amount of FVIIa, intrapulmonary administration of relatively small-dosed FVIIa could be an effective and reasonable treatment option for DAH in pediatric patients as well as in adults, requiring a prospective or randomized trial to verify the effect and standardize the FVIIa treatment. However, most importantly, many studies including our case series have suggested that instant hemostasis alone cannot ensure successful treatment outcome without successful treatment of primary disease or inflammation. We have experienced a refractory hemophagocytic lymphohistiocytosis (HLH) pediatric case presented with severe DAH (unpublished data). Although immediate and near complete hemostasis was achieved after intrapulmonary FVIIa instillations, the patients experienced multiple episodes of profound DAH and expired with sepsis and multiorgan failure, strongly implicating the importance of a multidisciplinary treatment approach for a successful outcome.

## Figures and Tables

**Table 1:** Etiologies of diffuse alveolar hemorrhage and treatment options.

Classification	Etiology		Nonspecific Findings	Specific Findings	Treatment	-
Immune-mediated	ANCA-associated vasculitis	Granulomatosis with polyangiitis (Wegener granulomatosis)	Prolonged PT/aPTT/PT INR	c-ANCA,	Immunosuppressants	Corticosteroids
		Microscopic polyangiitis	Thrombocytonia	p-ANCA (MPO-antibody)		Cyclophosphamide
		Eosinophilic granulomatosis with polyangiitis (Churg-Strauss syndrome)	Leukocytosis			Rituximab
	Anti-phospholipid antibody syndrome		Eosinophilia	APL, lupus anticoagulant, anti-CL, anti-β2GP1 antibody		Azathioprine
	SLE		Increased ESR	ANA, anti-dsDNA, anti-SM, anti-histone antibody		MMF
	RA		Hematuria	RF		IVIG
	Inflammatory myopathies		Patch diffuse alveolar infiltrates or			
	Henoch-Schönlein purpura			Anti-CL IgA antibody	Removal of autoantibodies	Plasmapheresis
	Ig A nephropathy		Air bronchogram on chest radiograph			
	Anti-GBM antibody syndrome (Goodpasture’s syndrome)			anti-GBM antibody	Procoagulation	Fresh frozen plasma
	Cryoglobulinemia					Platelet transfusion
	Behçet’s disease					Cryoprecipitates
	Hypocomplementemic urticarial vasculitis (anti-C1q vasculitis)					Vitamin K supplement
	Lung transplant rejection					TXA
	Drug-induced vasculitis					ACA
	Medications: warfarin, aspirin, amiodarone, phenytoin					FVIIa
	Idiopathic pulmonary capillaritis					Thrombin
Non-immune mediated	Coagulopathy					
	Non-cardiovascular disease				Withdrawal of offending medications	
	Infection	*Pseudomonas aeruginosa*				
		*Aspergillus* spp.			Broad-spectrum antibiotics	
		CMV and herpes pneumonitis				
	Diffuse alveolar damage	Radiation			Antifungal agents	
		Cytotoxic drugs				
		Acute respiratory distress syndrome			Antiviral therapy	
	Hematopoietic stem cell transplantation					
	Idiopathic pulmonary hemosiderosis				Diuresis	
	Cardiovascular disease	Pulmonary SOS		Kerley B lines on chest radiograph		
		Mitral stenosis			Cardiac medical optimization	
		Arteriovenous malformation				
		Pulmonary lymphangiomyomatosis				
		Pulmonary hypertension				
		Pulmonary capillary hemangiomatosis				
		Left ventricular dysfunction				

Abbreviations: ANA, anti-nuclear antibody; ANCA, anti-neutrophil cytoplasmic antibody; anti-β2GPA, anti-β-2 glycoprotein1 antibody; anti-CL antibody, anti-cardiolipin antibody; anti-dsDNA antibody, anti-double stranded DNA antibody; anti-MPO, anti-myeloperoxidase antibody; APL, anti-phospholipid antibody; anti-SM antibody; anti-smooth muscle antibody; C1q, complement 1q; c-ANCA, cytoplasmic-ANCA; CMV, cytomegalovirus; EACA, epsilon aminocaproic acid; ESR, erythrocyte sediment rate; FFP, fresh frozen plasma; GBM, glomerular basement membrane; INR, international normalized ratio; IPH, idiopathic pulmonary hemosiderosis; IVIG, intravenous immunoglobulin; MMF, mycophenolate mofetil; p-ANCA, perinuclear-ANCA; PT, prothrombin time; aPTT, a partial thromboplastin time; RA, rheumatoid arthritis; RF, rheumatoid factor; FVIIa, activated recombinant factor VII; SLE, systemic lupus erythematosus; SOS, sinusoidal obstruction syndrome; TXA, tranexamic acid.

**Table 2:** Published data on recombinant factor VIIa treatment for diffuse alveolar hemorrhage in adults.

References	Year	Patients (Sex/Age)	Previous History	Route	Dose of FVIIa	Additional Therapies	Outcome	TE Complication	Case of Death
Immune related									
Henke et al. [6]	2004	M/53	ANCA-vasculitis (*n* = 2), viral infection	IV	120 µg/kg × 3 doses	CS, CPM, MMF, plasmapheresis	CR	none	0
		M/25	SLE, APS, nephritis, pleural effusion	IV	90 µg/kg × 3 doses	CS	CR	none	0
Heslet et al. [5].	2006	M/63	Sarcoidosis, septic shock	IP	50 µg/kg × 1 dose	TXA, aprotinin, desmopressin	CR	none	1
		F/34	Wegener’s granulomatosis, Churg-Strauss vasculitis	IP	50 µg/kg × 1doses	TXA, aprotinin	CR	none	0
Dabar et al. [97]	2011	NA	ANCA-vasculitis	NA	90 µg/kg × 1 dose	none	CR	none	0
Mandal et al. [99]	2012	F/23	Microscopic polyangitis (pulmonary renal vasculitis), necrotizing glomerulonephritis	IV	90 µg/kg × 2 doses	MMF, plasmaphersis	CR	none	0
Esper et al. [82]	2014	F/37	SLE, Sjögren syndrome	IP	50 µg/kg × 1dose	CS, RTX	CR	none	0
Alabed et al. [100]	2014	F/37	SLE, lupus nephritis	IP	75 µg/kg × 1 dose	CS, CPM	CR	none	0
Khoulani et al. [101]	2014	F/51	NHL, lupus nephritis, cryoglobulinemia, bacterial pneumonia	IV	90 µg/kg × 3 doses	CS, RTX, plasmapheresis	NR	none	1
Pathak et al. [102]	2015	47 ± 19 years	ANCA vasculitis (*n* = 9)	IV	75 µg/kg × 4 doses	CS, CPM or RTX, IVIG Plasmapheresis	CR (9/9)	none	1/9
			Good pasture’s syndrome (*n* = 3)				CR (2/3), NR (1/3)	none	1/3
			SLE (*n* = 2)				CR (2/2)	none	0
			ITP (*n* = 1)				CR (1/1)	none	0
			Cryoglobulinemia (*n* = 1)				CR (1/1)	none	0
Baker et al. [75]	2016	F/23	SLE	IP	50 µg/kg × 1dose	CS, EACA, IVIG, CPM, RTX, plasmapheresis	PR	none	1
Diaz et al. [103]	2019	M/67	Wegener’s granulomatosis, bacterial pneumonia	IP	30 µg/kg × 1 dose	CS, desmopressin	CR	none	0
		F/61	SLE, pulmonary HTN, pneumonia	IP	50 µg/kg × 1dose	CS, plasmapheresis	CR	none	1
		F/22	Pulmonary sarcoidosis, pulmonary embolism, pneumonia	IP	50 µg/kg × 1dose	ECMO	CR	none	1
Non-immune related									
Meijer et al. [104]	2000	M/49	ALL, fungal pneumonia	IV	90 µg/kg × 1 dose	TXA, antifungal agents	CR	none	1
White et al. [105]	2001	M/ns	MDS, AML, Aspergillus pneumonia	IV	90 µg/kg × 4 doses	Antifungal agents	CR	none	0
Hicks et al. [106]	2002	F/35	AML, HCT, GVHD, fungal pneumonia	IV	90 µg/kg × 4 doses	CS, EACA, desmopressin	CR	none	0
Pastores et al. [4]	2003	M/48	NHL, HCT, GVHD	IV	90 µg/kg × 2 doses	CS	CR	none	0
Henke et al. [6]	2004	M/28	Acute leukemia, HCT	IV	120 µg/kg→180 µg/kg	CS	CR	none	1
Yildirim et al. [107]	2006	M/23	Pulmonary renal syndrome	IV	90 µg/kg × 3 doses	none	CR	none	0
Macdonald et al. [108]	2006	M/52	CAP	IV	90 µg/kg × 1 dose	none	CR	none	0
Heslet et al. [5]	2006	M/46	CLL, HCT, GVHD, CMV pneumonia	IV /IP	50 µg/kg IV × 3 doses/50 µg/kg IP × 2 doses	TXA, aprotinin	PR	none	1
		M/44	AML, pneumonia	IP	50 µg/kg × 1 dose	TXA, aprotinin	CR	none	0
		F/44	AIDS, C. difficile colitis, Pseudomonas pneumonia, CMV infection	IP	50 µg/kg × 1 doses	TXA, aprotinin	PR	none	1
		M/63	AML, HCT, GVHD, pneumonia	IP	50 µg/kg × 1 dose	TXA, aprotinin	CR	none	0
Shenoy et al. [109]	2007	F/NA	AML, HCT, pneumonia	IV	90 µg/kg × 2 doses	CS	CR	none	0
Gupta et al. [110]	2007	*n* = 24	HCT	NA	NA	CS (*n* = 24), desmopressin (*n* = 4), EACA (*n* = 3)	NA	none	NA
Estella et al. [98]	2008	F/39	AML, renal failure	IP	50 µg/kg × 1dose	None	CR	none	0
		M/46	IV drug abuse, hepatitis B and C, HIV, myocarditis, aspirin & clopidogrel	IP	50 µg/kg × 1dose	None	CR	none	0
Lau et al. [111]	2009	M/33	Cystic fibrosis	IV	90 µg/kg × 1 dose	BAE	CR	none	0
		M/22	Cystic fibrosis, liver failure, liver TPL, pneumonia	IV	90 µg/kg × 1 dose	BAE	CR	none	1
		F/23	Cystic fibrosis, lung infection, ARDS	IV	120 µg/kg × 2 doses	BAE	CR	none	1
		M/27	Cystic fibrosis, suppurative lung disease	IV	90 µgkg × 2 doses	BAE	PR/CR	none	0
Tatopoulous et al. [112]	2010	M/53	Leptospirosis, ARF, ARDS	IV	105 µg/kg × 1 dose	CS	CR	none	0
Dabar et al. [97]	2011	NA	Leukemia	NA	90 µg/kg × 1 dose	none	CR	none	0
Elinoff et al. [96]	2014	*n* = 23 (M:F =13:10), Age 36 (9–66) years	Diagnosis: ALL (*n* = 1), AML (*n* = 3), CLL (*n* = 2), HD (*n* = 1), AA (*n* = 6), MDS (*n* = 1), other (*n* = 5). Preceding conditions: HCT (*n* = 23), CMV (*n* = 14), aGVHD (*n* = 1), cGVHD (*n* = 8), DLI (*n* = 6), stem cell boost (*n* = 5), ARDS	IV	41 µg/kg × 3 doses, total dose 16mg, (4.8–37.6 mg)	CS (*n* = 23), Desmopressin (*n* = 8), EACA (*n* = 2), Estrogen (*n* = 1)	NA	44/43 episodes: blood clot obstruction an e-tube (*n* = 1), basilic vein thrombosis (*n* = 1). DIC (*n* = 2)	15/23
Pathak et al. [102]	2015	years	HCT (*n* = 7)	IV	75 µg/kg × 4 doses	CS, CPM or RTX, IVIG Plasmapheresis	CR (7/7)	none	6/7
Baker et al. [75]	2016	F/49	MDS, HCT	IP	30 µg/kg × 2 dose	CS, EACA	CR	none	0
		M/64	End-stage liver disease, clopidogrel treatment	IP	30 µg/kg × 2 dose	CS	CR	none	0
		F/68	Metastatic anal cell carcinoma, ARDS	IP	30 µg/kg × 2 dose	CS	CR	none	0
		F/23	HCT	IP	30 µg/kg × 1 dose	CS	CR	none	0
		F/84	Burn, inhaled injury	IP	60 µg/kg × 1 dose	CS, EACA	CR	none	0
Diaz et al. [103]	2019	F/46	Septic shock	IP	50 µg/kg × 1dose	none	CR	none	0
Shimizu et al. [113]	2020	M/68	Acute ischemic stroke, t-PA treatment	IV	75 µg/kg × 1dose	CS	CR	none	0
		M/54	Acute ischemic stroke, t-PA treatment	IP	75 µg/kg × 1dose	CS	CR	none	0

Abbreviations: AA, aplastic anemia; EACA, aminocaproic acid; aGVHD, acute graft-versus-host disease; AIDS, acquired immune deficiency syndrome; ALL, acute lymphocytic leukemia; AML, acute myeloid leukemia; ANCA, anti-neutrophil cytoplasmic antibody; APS, anti-phospholipid syndrome, ARDS, acute respiratory distress syndrome; BAE, bronchial artery embolization; CAP, community-acquired pneumonia; CLL, chronic lymphocytic leukemia; cGVHD, chronic graft-versus-host disease; CMV, cytomegalovirus; CR, complete response; CPM, cyclophosphamide; CS, corticosteroids; DLI, donor lymphocyte infusion; ECMO, extracorporeal membrane oxygenation; MDS, myeloid dysplastic syndrome; HCT, hematopoietic stem cell transplantation; HD, Hodgkin disease; HIV, human immune deficiency virus; HTN, hypertension; IP, intrapulmonary; ITP, idiopathic thrombocytopenic purpura; IV, intravenously; MMF, mycophenolate mofetil; NA, not available; NHL, non-Hodgkin lymphoma; NR, no response; SLE, systemic lupus erythematosus; PR, partial response; RTX, rituximab; TE, thromboembolic; TPL, transplantation.

**Table 3:** Published data on recombinant factor VIIa treatment for diffuse alveolar hemorrhage in children.

Reference	Year	Patients (Sex/Age)	Previous History	Route	Dose of FVIIa	Additional Therapies	Outcome	TE Complication	Case of Death
Immune-related									
Bafaquih et al. [121]	2015	3/8 patients, (M:F = 4:4), 2 (0.5–9) years	Connective tissue disorder (*n* = 3), respiratory infection (*n* = 3), MOF (*n* = 1)	IP	35–50 µg/kg × 3–6 dose	TXA	3CR	none	0
Congestive heart failure associated									
Veldman et al. [122]	2002	M/preterm	VLBW, RDS, PDA, IVH, PDA ligation	IV	200 µg/kg × 2 doses	none	CR	none	0
Olomu et al. [123]	2002	M/preterm	VLBW, RDS, PDA, sepsis, DIC, PIE	IV	50 µg/kg × 6 doses	none	CR	none	0
		F/preterm	VLBW, RDS, PDA, Sepsis, DIC	IV	50 µg/kg × 16 doses	none	CR	none	0
Leibovitch et al. [124]	2003	F/2 months	Down syndrome, CHD, cardiac surgery	IV	100 µg/kg × 4 doses	TXA	CR	none	0
Bafaquih et al. [121]	2015	1/8 patients	Cardiovascular disease, ARDS, infection	IP	35–50 µg/kg × 3–6 dose	TXA	1CR	none	0
Miscellaneous									
Blatt et al. [125]	2001	F/8 years	AML, HCT, HC	IV	270 µg/kg × 1 dose → 90 µg/kg × 28 doses	CS	PR	none	1
Cetin et al. [3]	2006	M/preterm	LBW, RDS, sepsis, DIC	IV	120 µg/kg × 3 doses	none	PR	none	0
Brady et al. [94]	2006	F/2 days	MMA, DIC, HD for hyperammonemia	IV	90 µg/kg × 2 doses		CR	none	1
		M/2 days	Pseudomonal sepsis	IV	90 µg/kg × 1 dose		CR	none	0
Grizelj et al. [126]	2006	NA/0 days	MAS, ventilator care	IV	170 µg/kg × 1 dose	EACA	CR	none	0
		NA/13 days	HLH, postsurgical resuscitation		130 µg/kg × 1 dose	EACA	CR	none	0
		NA/2 days	HLH, postsurgical resuscitation		222 µg/kg × 1 dose	EACA	CR	none	0
Young et al. [119]	2009	12 patients	NA	IV	90 µg/kg × 1 dose (range, 20.3–353 µg/kg)		4 CR, 5PR, 3NR	1 LV thrombus	NA
Bhat et al. [95]	2011	M/14 years	DSS, sepsis	IV	70 µg/kg × 1 dose	Anti-D	CR	none	0
		M/13 years	AML, TLS, ARF, acute pancreatitis	IV	90 µg/kg × 1 dose	none	PR	none	1
		F/9 years	Thalassemia, major, HCT, ARDS	IV	90 µg/kg × 1 dose	octreotide	CR	none	0
		F/13 years	ALL, febrile neutropenia, sepsis	IV	70 µg/kg × 1 dose	none	NR	none	1
		M/10 years	AML	IV	75 µg/kg × 1 dose	none	NR	none	1
Colin et al. [126]	2010	M/17 years	AML, pancytopenia, sepsis	IP	50 µg/kg × 1 dose	CS	CR	none	0
Larcombe et al. [99]	2014	M/2 years	AML, HCT, hepatic SOS, GVHD	IP	50 µg/kg × 1 dose	None	CR	ETT thrombus	0
Park et al. [81]	2015	F/11 years	MDS, HCT, HC, TMA	IP	60 µg/kg × 1 dose	CS, TXA, RTX	CR	none	1
		M/15 years	AML, DIC, cytarabine syndrome	IP	45 µg/kg × 1 dose	CS	CR	none	0
		M/6 years	T-LL, chickenpox infection, hepatic sinusoidal obstruction syndrome	IP	43 µg/kg × 1 dose	CS	CR	none	0
		M/14 years	AML, DIC	IP	52 µg/kg × 1 dose	CS	CR	none	0
		F/10 months	HLH, HCT, CMV infection, hepatic sinusoidal obstruction syndrome	IP	63 µg/kg × 1 dose	CS, TXA, RTX	CR	none	1
Bafaquih et al. [121]	2015	4/8 patients, (M:F = 4:4), 2 (0.5–9) years	Connective tissue disorder (*n* = 3), respiratory infection (*n* = 7), MOF (*n* = 1), cardiovascular disease (*n* = 1), ALL (*n* = 1), HLH (*n* = 1)	IP	35–50 µg/kg × 1 dose	TXA	2 CR, 2 PR	none	2/8
Idiopathic									
Bhat et al. [95]	2011	F/6 years	IPH, pneumothorax, and septic shock	IV	60 µg/kg × 1 dose	none	PR	none	1
Park et al. [81]	2015	F/11 years	IPH	IP	57 µg/kg × 1 dose	CS	CR	none	0

Abbreviations: ALL, acute lymphocytic leukemia; EACA, aminocaproic acid; AML, acute myeloid leukemia; ARDS, acute respiratory distress syndrome; ARF, acute renal failure; CHD, complex heart disease; CMV, cytomegalovirus; CR, complete response; CPM, cyclophosphamide; CS, corticosteroids; DIC, disseminated intravascular coagulation; DSS, Dengue shock syndrome; ETT, endotracheal tube; GVHD, graft-versus-host disease; HC, hemorrhagic cystitis; HCT, hematopoietic stem cell transplantation; HD, hemodialysis; HLH, hemophagocytic lymphohistiocytosis; HLHS, hypoplastic left heart syndrome; IP, intrapulmonary; IPH, idiopathic pulmonary hemosiderosis; IV, intravenously; IVH, intraventricular hemorrhage; LBW, low birth weight; LV, left ventricle; MAS, meconium aspiration syndrome; MDS, myelodysplastic syndrome; MMA, methylmalonic aciduria; NA, not available; NR, no response; PDA, patent ductus arteriosus; PIE, pulmonary interstitial emphysema; PR, partial response; RDS, respiratory distress syndrome; SOS, sinusoidal obstruction syndrome; TE, thromboembolic; T-LL, T-cell lymphoblastic lymphoma; TLS, tumor lysis syndrome; TMA, thrombotic microangiopathy; VLBW, very low birth weight.

## Data Availability

Data is contained within the article.

## Abbreviations

AAV	ANCA-associated vasculitis
ANA	anti-nuclear antibody
ANCA	anti-neutrophilic cytoplasmic antibody
APL	anti-phospholipid antibody
ARDS	Acute respiratory distress syndrome
BAL	bronchoalveolar lavage
β2GP	β-2glycoprotein1
CL	anti-cardiolipin antibody
CR	complete response
CT	Chest computed tomography
DAH	Diffuse alveolar hemorrhage
DAMPs	danger-associated molecular patterns
EACA	Epsilon-amino-caproic acid
ESRD	end-stage renal disease
ETT	Endotracheal tube
GBM	Glomerular basement membrane
GVHD	graft-versus-host disease
HCT	Hematopoietic stem cell transplantation
HLH	Hemophagocytic lymphohistiocytosis
ICU	Intensive care unit
IPH	Idiopathic pulmonary hemosiderosis
NET	Neutrophil extra-cellular traps
NR	No response
PEEP	Positive end-expiratory pressure
PR	Partial response
RDS	Respiratory distress syndrome
FVIIa	Recombinant factor VIIa
RF	Rheumatoid factor
TF	Tissue factor
TFPI	Tissue factor pathway inhibitor
TXA	Tranexamic acid
